# Yu-Ping-Feng Formula Exerts Antilung Cancer Effects by Remodeling the Tumor Microenvironment through Regulating Myeloid-Derived Suppressor Cells

**DOI:** 10.1155/2021/6624461

**Published:** 2021-04-20

**Authors:** Yuli Wang, Ningyang Sun, Yingbin Luo, Zhihong Fang, Yuan Fang, Jianhui Tian, Yongchun Yu, Jianchun Wu, Yan Li

**Affiliations:** ^1^Shanghai Municipal Hospital of Traditional Chinese Medicine, Shanghai University of Traditional Chinese Medicine, Shanghai 200071, China; ^2^Shanghai Longhua Hospital Affiliated to Shanghai University of Traditional Chinese Medicine, Shanghai 200032, China; ^3^Shanghai Chest Hospital Affiliated to Shanghai Jiaotong University, Shanghai 200030, China

## Abstract

Yu-Ping-Feng (YPF) formula is a classical prescription used for enhancing the body's immunity function in traditional Chinese medicine (TCM). In clinical practice, the YPF formula has been reported to exhibit antilung cancer and immunomodulatory effect. However, the relationship between them remains unclear. The present study aimed to investigate the antilung cancer effect of the YPF formula and its immune-related mechanisms. The C57BL/6 tumor-bearing mice model was established and randomly divided into the YPF group and the control group. Tumor volume, spleen weight, and survival in both groups were measured and evaluated during 28 days of consecutive intervention. Flow cytometry was used to detect the proportion of immune cell subsets. Myeloid-derived suppressor cells (MDSCs) were induced in vitro from bone marrow cells. After intervention by the YPF formula, CCK-8 and flow cytometry analyses were performed to detect proliferation and apoptosis of MDSCs. A coculture system containing T cells and MDSCs was established to further study the role of MDSCs in the regulation of T-cell subsets proportion by the YPF formula. The expressions of MDSCs-related genes and proteins were detected by RT-PCR and Western blotting. The results showed the YPF formula inhibited tumor growth, reduced spleen weight, and prolonged the survival of mice. Besides, the proportions of MDSCs subsets and Regulatory *T* (Treg) in the YPF group decreased, whereas those of CD_4_^+^T and CD_8_^+^T increased both in vitro and in vivo. CCK-8 and flow cytometry demonstrated that the YPF formula could inhibit proliferation and promote apoptosis of MDSCs. The coculture experiments further confirmed that MDSCs served a critical role in regulating the tumor microenvironment by the YPF formula. RT-PCR and Western blotting indicated that the levels of MDSCs' activation and proliferation-related proteins and genes were downregulated in the YPF group. Therefore, our results demonstrated that the YPF formula could promote apoptosis and inhibit the proliferation of MDSCs. As a result, the negative regulatory effect on the positive immune cells induced by MDSCs was weakened, thus achieving the antilung cancer effect by remodeling the tumor microenvironment.

## 1. Introduction

Lung cancer is the leading cause of cancer morbidity and mortality worldwide [1]. In China, according to relevant statistical results, there were about 787,000 new cases of lung cancer and about 631,000 deaths resulting from lung cancer [2]. In recent decades, apart from classic treatment strategies, several novel targeted drugs for patients with EGFR mutated or ALK rearranged have been introduced to the treatment of lung cancer [3]. Furthermore, some epigenetic alternations such as RNA methylation, histone modifications, or other molecular events have provided clues to identify novel therapeutic targets and early detection of cancers [4, 5]. However, the five-year overall survival rate of lung cancer remains at a low level, which is around 16.1% in China [6]. The clinical efficacy of killing tumor cells alone is still far from satisfactory. With the deepening understanding of “soil-seed” theory, tumor microenvironment (TME) that is closely related to the tumor genesis and development has achieved more attention [7]. More and more research has demonstrated that tumor cells were assisted with a series of immunosuppressive cells in the TME to escape from immune surveillance. Besides, excessive infiltration of inflammatory cytokines could promote the proliferation, migration, and differentiation of the tumor cells by activating tumor-associated signaling cascades. Therefore, targeting the dynamic TME may provide an effective therapeutic strategy for cancer [8]. For instance, Treg in the TME is one of the critical factors in the formation of tumor immunosuppression, which is also a key barrier to successful tumor immunotherapy [9]. Related research demonstrated that Treg cells were significantly increased in acute myeloid leukemia (AML), and downregulating the level of CD_25_^+^FOXP_3_^+^Treg cells in the TME contributed to clinical benefit in patients with refractory AML. Besides, Treg molecular markers were significantly predictive of survival in AML patients [10]. Apart from Treg cells, MDSCs are another typical representative. Previous research demonstrated that large infiltration of MDSCs in the TME was closely related to the poor prognosis of lung cancer [11]. Thus, targeting the phenotype and function of MDSCs in the TME may be one of the potential treatment strategies for lung cancer [12].

MDSCs, as one of the most important immune suppressive cells in the TME, possess complex phenotypes. In mice, the phenotype of MDSCs is Gr-1^+^CD11b^+^. According to the difference in epitopes, MDSCs can be further divided into monocytic MDSCs (Gr-1^+^CD11b^+^Ly6G^−^Ly6C^hi^) and granulocytic MDSCs (Gr-1^+^CD11b^+^Ly6G^+^Ly6C^lo^) [13, 14]. Existing research has proven chronic inflammatory and the establishment of TME could promote the generation and activation of MDSCs. As a result, the activation of MDSCs could in turn suppress the immune response and promote tumor progression [15]. For example, MDSCs could interfere with the immunity function of CD_8_^+^T lymphocytes and promote the activation of Treg cells to mediate immune tolerization [16, 17]. MDSCs could also promote the polarization of macrophages toward the M2 phenotype and form into tumor-associated macrophages (TAMs) with negative immune regulatory effects [18]. Moreover, MDSCs could secrete TGF-*β* to inhibit the natural killer (NK) cells' function [19]. Therefore, MDSCs are one of the key factors which can trigger a vicious circle of the immune response in the TME.

The YPF formula is a classical TCM prescription that was invented by *Zhu Danxi* during the *Ming Dynasty* of China. It is composed of Astragali Radix (*Huang-Qi*), Atractylodis Macrocephalae Rhizoma (*Bai-Zhu*), and Saposhnikoviae Radix (*Fang-Feng*) in a weight ratio of 2: 2: 1. The YPF formula has been applied in clinical practice for the prevention and treatment of cold and flu for several centuries. Nowadays, because of its unique immune regulation effect, it has been widely used in many immune-related diseases such as allergic rhinitis [20], asthma [21], chronic kidney disease [22], malignant tumors [23], and so on. A clinical study showed that the YPF formula could improve the immune function and the life quality of lung cancer patients undergoing chemotherapy [24]. Since there is proven antitumor effect of the YPF formula in our previous studies and its immunomodulatory effect [25], we try to elucidate the potential antilung cancer mechanism of the YPF formula from the perspective of modulating the tumor immune microenvironment.

## 2. Materials and Methods

### 2.1. Drug Preparation

All granules for the in vivo experiments were purchased from Jiangyin Tianjiang Pharmaceutical Co. Ltd. The clinical drug dose and medicinal part of each herb are listed in Table 1. The equivalent dose was calculated according to the clinical human dose (45 g per day) and the surface area ratio of the human to the animal. Consequently, the concentration of the YPF formula was diluted to 2340 mg/mL and the dose of the YPF formula administered per mouse was 200 *μ*L for the in vivo experiment. After filtering through 0.4 *μ*m and 0.22 *μ*m filters successively, the granules were stored at −20°C until used. The composition and quality control of the granules were performed by high performance liquid chromatography (HPLC). Referring to the relevant quality control standards in the Chinese Pharmacopoeia (China Pharmacopoeia Committee, 2015), Astragaloside IV, Atractylenolide II, Prim-O-glucosylcimifugin, and 5-O-methylvisammioside were used as standard controls of the above three herbs, respectively, as shown in Supplementary Figure 1.

Lyophilized powder of the YPF formula was applied in the in vitro study. The raw herbs of Astragali Radix (Huang-Qi), Atractylodis Macrocephalae Rhizoma (Bai-Zhu), and Saposhnikoviae Radix (Fang-Feng) were purchased from the pharmacy of Shanghai Municipal Hospital of TCM. The quality of the herbs was identified by Professor Haiqing Zhu. The specific drug dose of the YPF formula was 18 g:18 g:9 g (Huang-Qi: Bai-Zhu: Fang-Feng). The extract of the YPF formula was diluted 10 times in distilled water and heated for three hours under continuous stirring at 100°C. The process was repeated twice and the extract was centrifuged at 1500 g. The supernatant was collected and evaporated at 70°C until the semisolid was formed. Triethanolamine was used as a neutralizer to regulate the pH value between 6 and 8 of the lyophilized powder. The mixture concentration was diluted to 1 g/mL by DMEM and stored at −20°C until use. A sample of the YPF formula was kept in our laboratory for future reference.

### 2.2. Reagents

The DMEM medium, PBS medium, and 10% fetal bovine serum were purchased from Gibco Life Technologies (Grand Island, NY, USA). The tumor dissociation kit and spleen dissociation kit were obtained from Miltenyi Biotec (San Diego, CA, USA). Anti-mouse PerCP/Cy5.5 CD_3_, anti-mouse FITC CD_4_, anti-mouse PE CD_8_, anti-mouse APC CD_25_, anti-mouse FITC Gr-1, anti-mouse PE CD_11b_, anti-mouse PerCP/Cy5.5 Ly6c, and anti-mouse APC Ly6g were all purchased from Biolegend (San Diego, CA, USA). 5%BSA, paraformaldehyde, electrophoresis solution, transfer solution, CCK-8 kit, RIPA were all provided by Beyotime Biotech (Beijing, China). The SDS-PAGs (Sodium Dodecyl Sulfate-Polyacrylamide Gels) were bought from Dakewe Biotech (Shenzhen, China). RT-PCR primers were designed and provided by Sangon Biotech (Shanghai, China). Trizol reagent, Tween-20, and 20 × TBS buffer were obtained from Thermo Scientific (Rockford, IL). Antibodies for Western blot were all purchased from Cell Signaling Technology (MA, USA). An ECL Kit was provided by Tanon Biotech (Shanghai, China).

### 2.3. Cell Lines and Cell Culture

LLC (Lewis lung cancer) cells were purchased from the cell bank of Shanghai Institute of Life Sciences, Chinese Academy of Sciences (Shanghai, China). The LLC cells were cultured in the DMEM medium, supplemented with 100U/L penicillin, 0.1 mg/mL streptomycin, and 10% fetal bovine serum. Cells were maintained at 37°C in a cell incubator with an atmosphere of 5% CO_2_.

### 2.4. Animal Treated with the YPF Formula

Five-week-old C57BL/6 mice (male, 18–22 g) were purchased from Slack Experimental Animals Co. Ltd. of the Chinese Academy of Sciences (Shanghai, China). Mice were maintained in the Animal Experimental Center of Shanghai Municipal Hospital of TCM. The environment was strictly controlled at the temperature of 20–24°C, the humidity of 50%–60%, as well as the 12 h/12 h light-dark cycle. Food and water were free to access for mice and provided by the animal experiment center. Ten mice were randomly divided into the control group and the YPF group (five per group). Logarithmic growth LLC cells were digested and counted. Each mouse was subcutaneously inoculated with 1 × 10^6^ LLC cells into the right flanks. 24 hours after inoculation, mice in the YPF group were treated by oral gavage with 200 *μ*L of the YPF formula, while the control group mice were given the same volume of normal saline. The volumes of tumor xenografts were measured with a Vernier caliper every 7 days, and the survival of mice in each group was recorded. Finally, the survival curves were drawn according to the Kaplan-Meier method. All animal experiments were approved by the Institutional Animal Care and Use Committee of Shanghai Municipal Hospital of Traditional Chinese Medicine (Shanghai, China). The approval number for the present study is #dw2020005.

### 2.5. Preparation of Spleen and Tumor Tissue Single-Cell Suspensions

For spleen single-cell suspension preparation, we minced the spleen tissues by a scissor and put them into a C tube of gentle MACS. Then, the corresponding enzyme solution was prepared and added. After ground by the gentleMACS machine and filtered through a 30 *μ*m filter, the cells were centrifuged at 300 g for 10 minutes and the supernatant was discarded. 3 mL erythrocyte lysate was then added to dissolve red blood cells. Finally, the cells were resuspended with PBS solution, counted, and set aside for use. The single-cell suspension of tumor tissues was prepared by a similar method. 0.04–0.1 g tumor tissues were weighed and ground, after filtering through a 70 *μ*m filter; the cells were centrifuged at 300 g for 10 minutes. Discard the supernatant and add 3 mL erythrocyte lysate. Finally, the cells were resuspended by PBS solution for later use.

### 2.6. Establishment and Induction of Bone Marrow-Derived MDSCs

After sacrificing the mice by cervical dislocation, the 1640 medium was used to rinse the bone marrow cavity of the tibia and femur to obtain the bone marrow. The bone marrow sample was filtered through a 45 *μ*m strainer to remove the debris. Then, erythrocyte lysate was added and stood still for 3–4 minutes to eliminate red blood cells. The reaction mixture was filtered again and centrifuged at 4300 rpm for 5 minutes. The precipitated bone marrow cells were collected and the supernatant was discarded. Finally, MDSCs induction was carried out under the condition of IL-6 (20 ng/mL) and GM-CSF (20 ng/mL). MDSCs with a purity of 88.3% were eventually obtained as determined by flow cytometry (see Supplementary Figure 2).

### 2.7. Establishment of the Coculture System Containing MDSCs and T Lymphocytes

T lymphocytes were isolated by the method of Ficoll density gradient centrifugation. The spleen was thoroughly minced and ground on a 100-mesh filter. The spleen was washed with PBS and the cell suspension was collected. Ficoll was then added to the cell suspension in a 1 : 1 ratio and centrifuged at 2000 rpm for 20 minutes. The T lymphocyte was aspirated in the narrow zone at the junction of the two layers, and PBS was added, centrifuged at 4°C (2000 rpm for 10 min), and resuspended. Finally, the MDSCs and T lymphocytes were mixed in a 1 : 1 ratio (3 × 10^5^) to establish a coculture system.

### 2.8. Detection of Cell Apoptosis by Flow Cytometric Analysis

Cell apoptosis was detected using the Annexin V-FITC/PI Apoptosis Detection Kit according to the manufacturer's protocol. About 1 × 10^5^ bone marrow cells were collected and centrifuged at 300 g for 5 minutes. 5 *μ*L Annexin V-FITC and propidium iodide (PI) was added and incubated at 4°C for 30 minutes in darkness. The cell apoptosis rate was detected by flow cytometric (Beckman Coulter, USA). Survival cells were both negative in Annexin V-FITC and PI, while the late apoptosis cells were both positive in V-FITC and PI. For early apoptosis cells, they were positive in Annexin V-FITC, negative in PI. In contrast, for dead cells, they were negative in Annexin V-FITC, positive in PI.

### 2.9. RT-PCR

RT-PCR assay was performed to detect STAT3, iNOS, and Arg-1 mRNA expression levels in vivo and in vitro. TRIzol reagent was used to extract the total RNA according to the manufacturer's instructions. Reverse transcription was carried out to acquire cDNA using a High-Capacity cDNA Reverse Transcription Kit (Takara Bio, China). SYBR green real-time PCR super mix was used for PCR amplification. All genes were amplified under the standard PCR conditions with 37 cycles, the annealing step lasted for 20 seconds at 60°C, and extension step lasted for 60 seconds at 72°C. The primer sequences of the genes were designed and displayed as followed: iNOS: F: AGATTCCGTCCATCAAGT; R: CAGTCCTCGGGTAGTCAA; Arg-1: F: AGTCAGTCCCTGGCTTAT; R: AAGACAGCAGAGGAGGTG; STAT3: F: CCAGCAACCTGACTTTCG; R: TTCAGACCCGCCAACAAA; GAPDH: GTGGAGATTGTTGCCATCAACGA; R: CCCATTCTCGGCCTTGACTGT. The expression level of the GAPDH gene served as endogenous control, and the 2^−△△Ct^ value was used to qualify the relative gene expression levels.

### 2.10. Western Blot Assay

The samples were completely lysed and centrifuged, and the supernatant was collected for quantitative protein analysis. About 50 *μ*g proteins were added to each well of the SDS-PAG. By SDS-PAG electrophoresis (SDS-PAGE). Protein samples were concentrated, separated, and transferred to a polyvinylidene fluoride (PVDF) membrane. The membranes were blocked with 5% BSA for 2 hours at room temperature. Then, the following primary antibodies (see Supplementary Table 1) were added and kept in a wet box overnight at 4°C in darkness. After incubating the PVDF membranes with the secondary antibody (dilution 1 : 1000) the following morning for one hour, the protein bands were detected with an ECL kit and scanned by a Gel Image system ver4.0 (Tanan, China). Quantitative analysis of the protein bands was carried out using the ImageJ 6.0 software, and the results were normalized to *β*-actin bands.

### 2.11. Statistical Analysis

SPSS 25.0 for Windows (SPSS Inc, Chicago, IL, USA) and GraphPad Prism 8.0 (GraphPad Software Inc, California, USA) were used for statistical analysis and result representation. All continuous data were expressed as the mean ± standard deviation. Comparisons between the two groups were performed by Student's *t*-test or the Rank Sum test. A *P* value of less than 0.05 was considered to be statistically significant.

## 3. Results

### 3.1. The YPF Formula Suppressed LLC Xenograft Tumor Growth and Prolonged the Survival of Tumor-Bearing Mice

**Figure 1:**
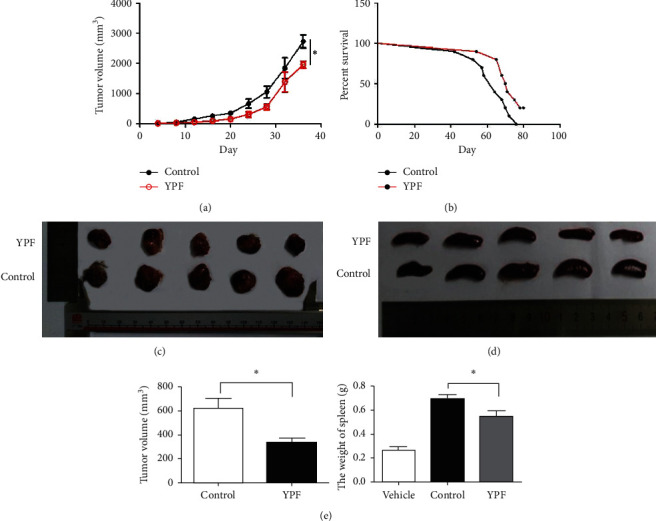
The YPF formula suppressed LLC xenograft tumor growth and prolonged the survival of tumor-bearing mice. All values are expressed as mean ± SD, *n* = 5 for each group. (a) Tumor volumes were measured. (b) Mice were monitored for survival. (c) Representative picture of tumors in both groups. (d) Representative picture of spleens in both groups. (e) Tumor volumes and spleen weights were measured at 28 d after the intervention. *∗P* < 0.05 vs. control.

To explore the antitumor effect of the YPF formula, LLC cells (1 × 10^6^) were inoculated subcutaneously into the right flanks of C57BL/6 mice (*n* = 5 per group). After tumors were palpable, mice were divided into two groups randomly. Another vehicle group was set up, and the spleen sizes of mice in the group were used as controls. The YPF group was given the YPF formula by oral gavage every day, while the control group was given the same volume of normal saline for 4 weeks. Results revealed increased survival time and decreased tumor volume in the YPF group mice compared to the control group, as shown in Figure 1. Interestingly, we also found that, after the intervention of the YPF formula, the spleen weights of the tumor-bearing mice were significantly lower than those in the control group, which were much closer to the vehicle group.

### 3.2. The YPF Formula Regulated the Proportion of Immune Cells in the Spleen and Tumor Tissue

**Figure 2:**
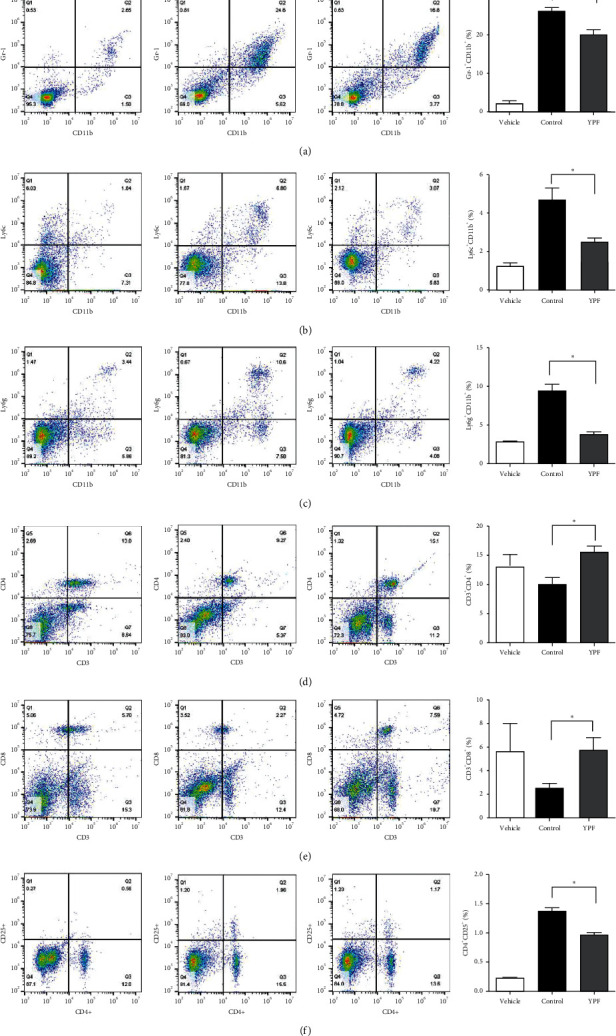
Effects of the YPF formula on the proportion of immune cells in the spleen. (a) The proportion of MDSCs (Gr-1^+^CD11b^+^). (b) The proportion of monocytic MDSCs (Gr-1^+^CD11b^+^Ly6G^−^Ly6C^hi^). (c) The proportion of granulocytic MDSCs (Gr-1^+^CD11b^+^Ly6G^+^Ly6C^lo^). (d) The proportion of CD_3_^+^CD_4_^+^T lymphocytes. (e) The proportion of CD_3_^+^CD_8_^+^T lymphocytes. (f) The proportion of CD_4_^+^CD_25_^+^T lymphocytes (Treg). *∗P* < 0.05 vs. control.

**Figure 3:**
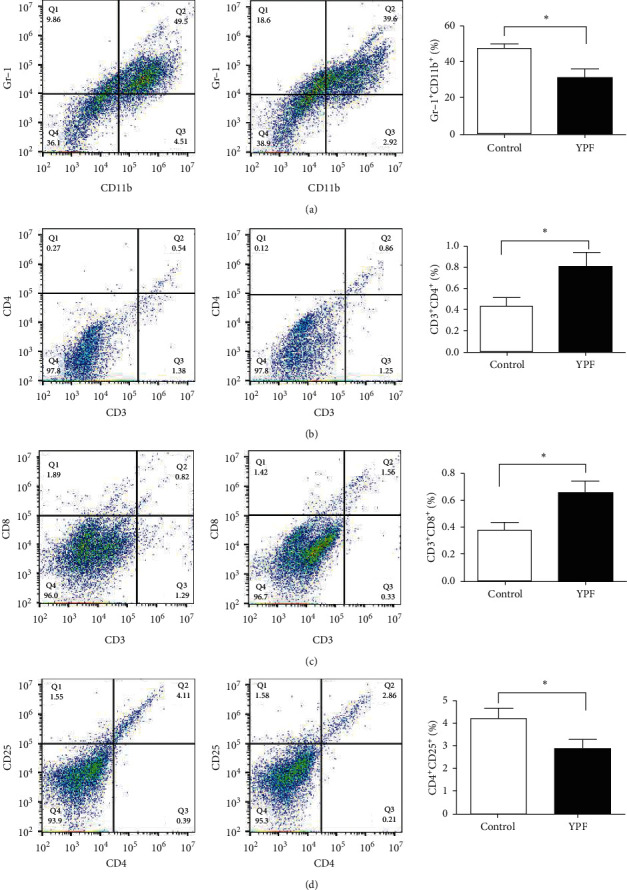
Effects of the YPF formula on the proportion of immune cells in tumor tissues. (a) The proportion of MDSCs (Gr-1^+^ CD11b^+^). (b) The proportion of CD_3_^+^CD_4_^+^T lymphocytes. (c) The proportion of CD_3_^+^CD_8_^+^T lymphocytes. (d) The proportion of CD_4_^+^CD_25_^+^T lymphocytes (Treg). *∗P* < 0.05 vs. control.

Since the potential immunomodulatory effects exhibited by the YPF formula, we prepared the single-cell suspensions of the tumor tissues and spleens from tumor-bearing mice to further explore the specific immunomodulatory mechanism. Results showed that, compared with the control group, the proportions of both granulocyte-like and monocyte-like MDSCs in mice spleen and tumor tissue were significantly decreased in the YPF group. For T-cell subsets, the proportions of CD_4_^+^T and CD_8_^+^T in the YPF group increased, whereas the proportion of Treg decreased significantly in the YPF group, as shown in Figures 2 and 3. These results indicated that the YPF formula could positively regulate the immune response of tumor-bearing mice, which was manifested as a decrease in the proportion of negative immune cells (MDSCs subsets and Tregs), as well as an increase in the proportion of positive immune cells (CD_4_^+^ and CD_8_^+^T cells) in the tumor microenvironment.

### 3.3. The YPF Formula Promoted the Apoptosis of MDSCs and Inhibited Its Proliferation

**Figure 4:**
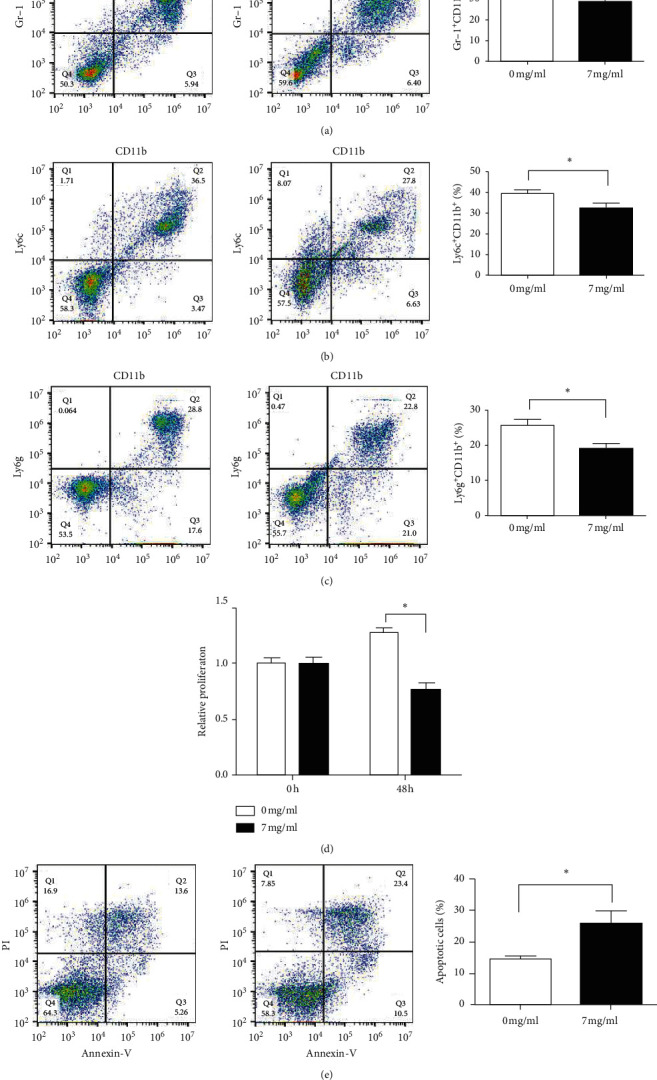
Effects of the YPF formula on proliferation and apoptosis of MDSCs and its subsets. (a) Proportion of MDSCs (Gr-1^+^ CD11b^+^). (b) The proportion of monocytic MDSCs (Gr-1^+^CD11b^+^Ly6G^−^Ly6C^hi^). (c) The proportion of granulocytic MDSCs (Gr-1^+^CD11b^+^Ly6G^+^Ly6C^lo^). (d) The proliferation of MDSCs was detected by the CCK-8 assay. (e) Apoptotic cells were routinely detected by the Annexin V/PI staining method. The proportion of apoptotic cells is shown in the lower right quadrant, and the cells shown in the upper right quadrant are necrotic cells or cells with secondary necrosis after apoptosis. The concentration of the YPF formula was 0 mg/mL for the control group and 7 mg/mL for the YPF group. *∗P* < 0.05 vs. control.

For in vitro experiment, the concentration of the YPF formula was set as 7 mg/mL according to the IC50 value determined in our previous research. The in vitro experiments also confirmed that, after the YPF formula's intervention, the total MDSCs and its subsets in the YPF group were significantly reduced. As shown in Figure 4, the CCK-8 assay demonstrated that cell proliferation of MDSCs was significantly inhibited in the YPF group. Flow cytometric detection indicated that, compared with the control group, the proportion of apoptotic cells in the YPF group increased significantly. These experimental results revealed that the YPF formula could promote the apoptosis of MDSCs and inhibited its proliferation and thus played a role in regulating the tumor microenvironment.

### 3.4. The YPF Formula Regulated the Proportion of T-Cell Subsets in the Coculture System by Inhibiting MDSCs

**Figure 5:**
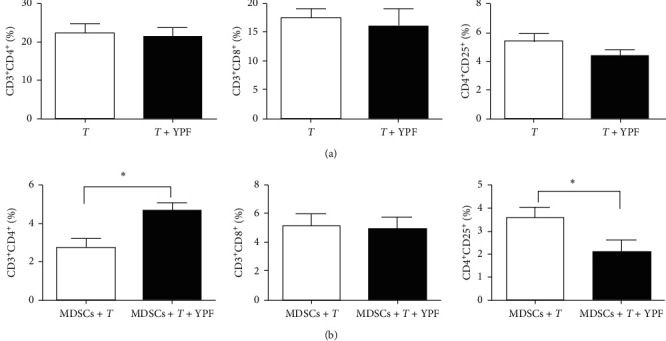
The YPF formula regulated the proportion of T-cell subsets in the coculture system by inhibiting MDSCs. (a) Effect of the YPF formula on the proportion of T-cell subsets. “*T*” represents the control group; “*T* + YPF” represents the experimental group. (b) Effect of the YPF formula on the proportion of T-cell subsets in the coculture system. “MDSCs + *T*” represents the control group; “MDSCs + *T* + YPF” represents the experimental group. *∗P* < 0.05 vs. control.

To further investigate the role of MDSCs in the regulation of T-cell subsets proportion by the YPF formula, we established a coculture system of T lymphocytes and MDSCs. As shown in Figure 5, for T-lymphocyte suspension, the YPF formula failed to produce a significant change in the proportion of T-cell subsets, while, in the coculture system of T lymphocytes and MDSCs, results showed that the intervention of the YPF formula significantly increased the proportion of CD_4_^+^ T lymphocytes and decreased the proportion of Treg. The results of the experiments presented above indicated that the effect of the YPF formula on the T-cell subset proportions in the coculture system may be realized by inhibiting MDSCs.

### 3.5. The YPF Formula Downregulated the Expression Levels of MDSCs Immunosuppressive-Related Genes and Proliferation-Related Proteins

To verify the effect of the YPF formula on MDSCs related immunosuppressive genes, the RT-PCR assay was conducted. Results showed that, after intervention by the YPF formula, the levels of Arg-1, iNOS, and STAT3 were significantly decreased in vivo and in vitro. These genes were considered to be closely involved in the proliferation and activation of MDSCs.

**Figure 6:**
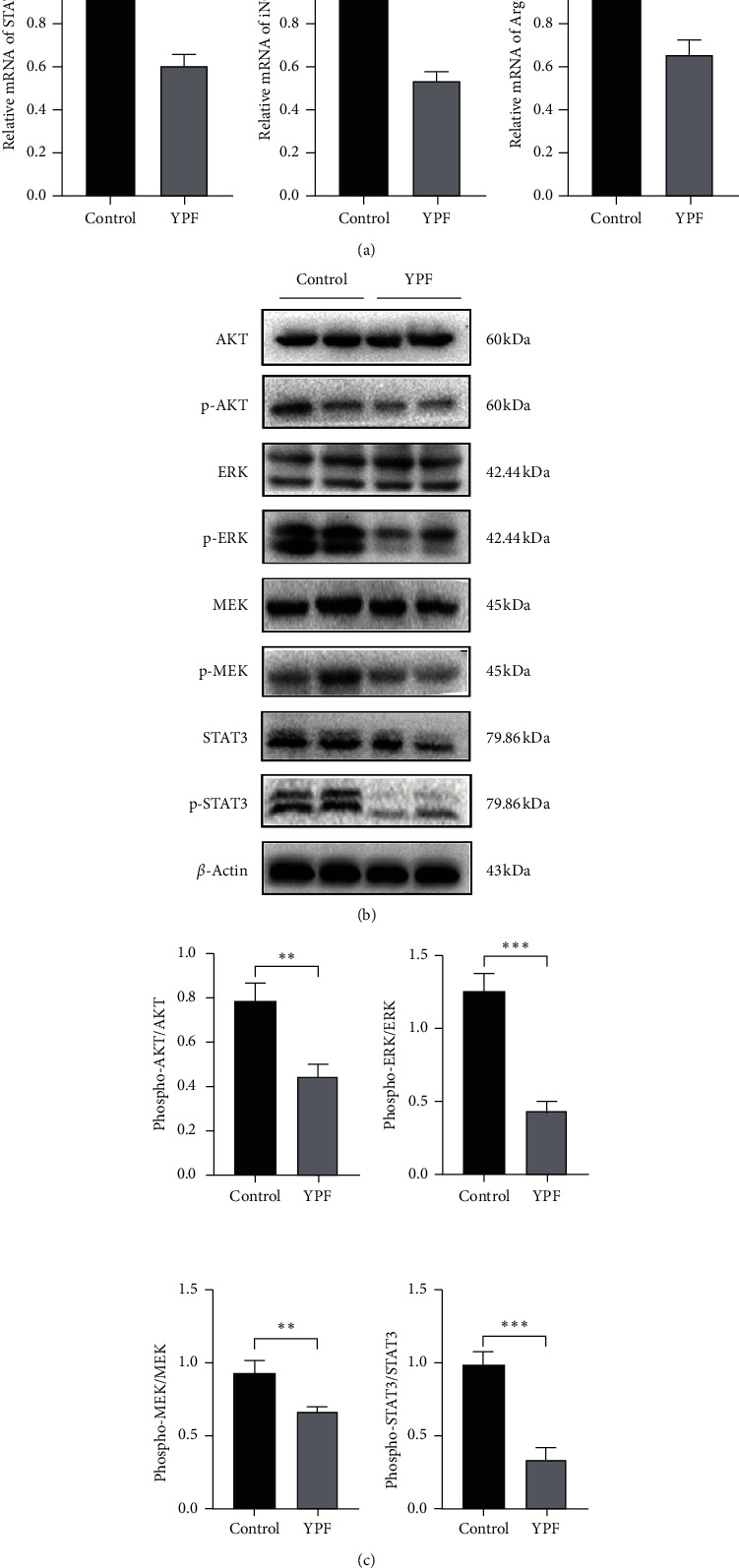
The YPF formula downregulated the expression levels of MDSCs immunosuppressive-related genes and proliferation-related proteins in vivo. (a) RT-PCR was used to detect the expression levels of STAT3, iNOS, and Arg-1 mRNA in the tumor tissues. (b) Western blot was used to investigate the expression of MDSCs proliferation-related proteins p-STAT3, p-AKT, p-MEK, and p-ERK in the tumor tissues. All data were expressed as the mean ± SD from at least three independent experiments. *∗P* < 0.05, *∗∗P* < 0.01, and *∗∗∗P* < 0.001.

**Figure 7:**
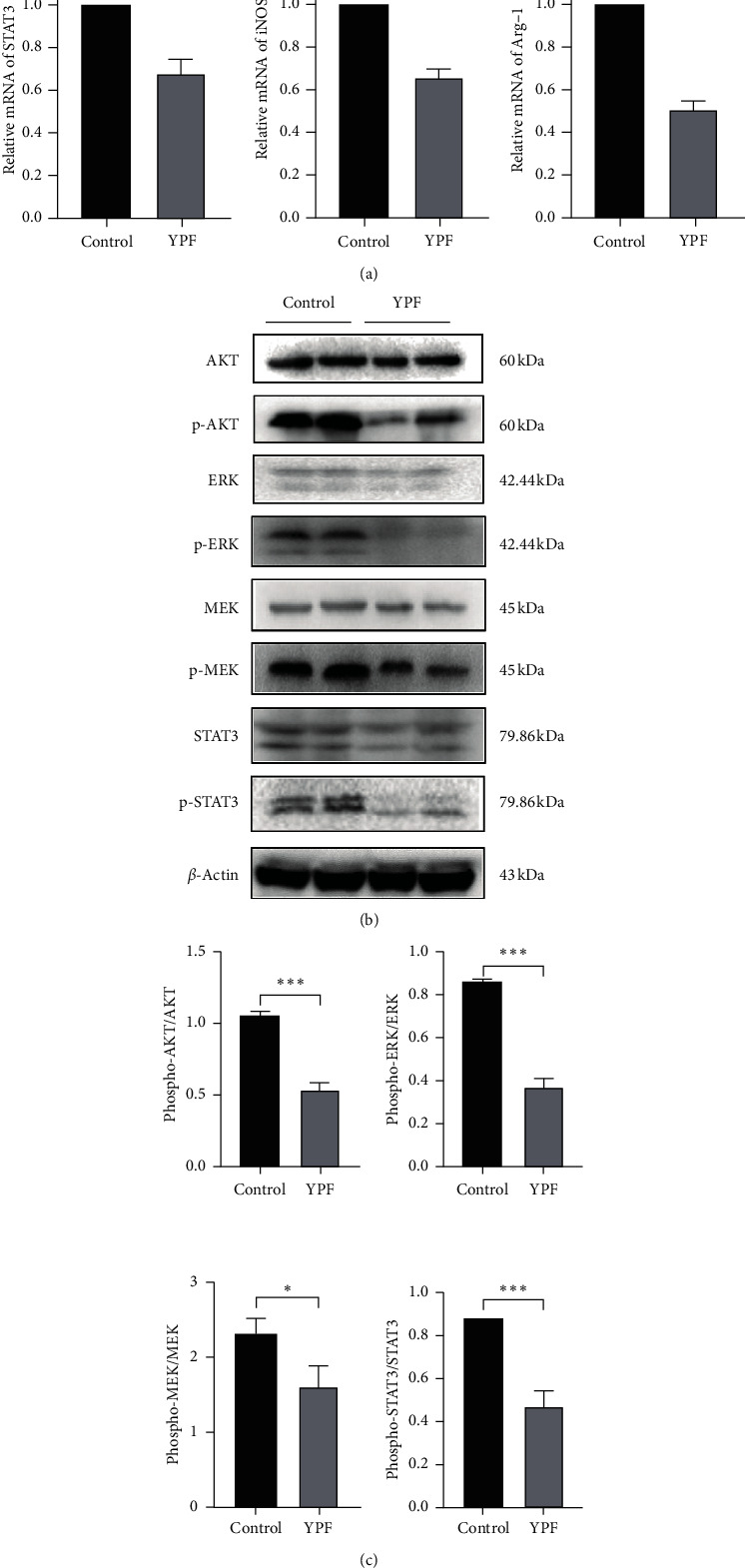
The YPF formula downregulated the expression levels of MDSCs immunosuppressive-related genes and proliferation-related proteins in vitro. (a) RT-PCR was used to detect the expression levels of STAT3, iNOS, and Arg-1 mRNA in vitro. (b) Western blot was used to investigate the expression of MDSCs proliferation-related proteins p-STAT3, p-AKT, p-MEK, and p-ERK in vitro. All data were expressed as the mean ± SD from at least three independent experiments. *∗P* < 0.05, *∗∗P* < 0.01, and *∗∗∗P* < 0.001.

Western blot analysis confirmed that the expression levels of phospho-AKT, phospho-MEK, phospho-ERK, and phospho-STAT3 protein in the YPF group were significantly decreased. It indicated that the YPF formula could downregulate the expression levels of MDSCs proliferation-related proteins, as shown in Figures 6 and 7.

## 4. Discussion

Lung cancer is one of the most common malignancies in China and even worldwide, which is becoming the main killer of human health, due to its high global incidence and mortality. In recent years, due to the promotion of smoking cessation education, early screening work, and the progress of treatment fields (such as targeted therapy, immunotherapy, etc.), the mortality rate of lung cancer has shown a rapid trend of decline [26]. However, the current five-year survival rate remains low. As research progresses, current research is no longer limited to the traditional strategies to kill tumor cells; the concept of the tumor microenvironment has gradually attracted the attention of researchers.

The tumor microenvironment is a unique complex network environment in the process of tumor progression, which is not only the site of tumor cells but also including a series of cells that contribute to the immunosuppressive properties of the tumor environment, such as MDSCs. MDSCs, as precursors of dendritic cells, macrophages, and granulocytes, are heterogeneous cell populations derived from bone marrow progenitors and immature myelocytes [27]. Under normal circumstances, these cells can differentiate into mature cells. However, in the tumor environment, these cells are not fully differentiated. Instead, they are recruited from the bone marrow to the periphery blood and activated, playing an important role in suppressing antitumor immunity. Therefore, relevant studies on MDSCs may open up new ideas for remodeling the tumor environment and inhibiting the occurrence and metastasis of tumors.

YPF formula, as a classic prescription of TCM, was first seen in Danxi's experiential therapy in Ming Dynasty. Pharmacological studies indicated that the YPF formula could play an immunomodulatory role by enhancing the activation, proliferation, and phagocytosis of macrophages. Besides, it could also regulate the anti-inflammatory activity of T lymphocytes and enhance the phagocytic activity of NK cells [25, 28, 29]. In vitro studies have also demonstrated that the YPF formula has broad-spectrum antitumor effects on various kinds of tumor-bearing mice. Therefore, based on the immunomodulatory function of the YPF formula, it makes it feasible for the YPF formula to play the role of antitumor.

In the present study, it was found that the YPF formula could inhibit tumor growth in Lewis lung carcinoma tumor-bearing mice and prolong survival. After intervention by the YPF formula, it also brought about the changes in the downregulation of the proportion of MDSCs and Treg cells, as well as the upregulation of CD_4_^+^*T* and CD_8_^+^ T lymphocytes. Furthermore, it was worth mentioning that the spleens of mice in the YPF group were significantly smaller than those in the control group. Based on the above facts, it can be inferred that the antitumor effect of the YPF formula is closely related to immunomodulation. However, the specific mechanisms involved in the process remained unknown.

Previous researches have shown that MDSCs exerted the immunosuppression effect in multiple ways. First of all, MDSCs suppressed the proliferation and activation of CD_4_^+^*T* and CD_8_^+^ T lymphocytes in an antigen-specific manner limited or not limited by major histocompatibility complex, which significantly reduced the body's antitumor immunity. Besides, MDSCs can inhibit T-lymphocyte proliferation and activation by overexpressing arginase 1, depleting two essential amino acids (cysteine and arginine) crucial for T-lymphocyte synthesis [30]. Mediated through the generation of ROS and NO, MDSCs can downregulate the expression of the CD3*ζ* chain in T lymphocytes, thus inhibiting the activation of T lymphocytes [31]. Moreover, MDSCs can also secrete a series of cytokines, such as IL-10 and TGF-*β*, and present tumor-associated antigens, to induce the proliferation of Treg cells [32, 33]. The present study has demonstrated that the regulatory effect of the YPF formula on MDSCs and T lymphocytes may be one of the critical ways to remodel the immune microenvironment and serve as an antitumor role. The establishment of the coculture system in vitro and functional experiments further confirmed that the regulation of T-lymphocyte subsets by the YPF formula was related to the promotion of apoptosis and inhibition of proliferation of MDSCs. The PCR results showed that the mRNA expression levels of iNOS and Arg-1 were significantly decreased in the YPF group. This suggested that reducing oxidative stress and promoting regulation of T-lymphocyte proliferation may be the subsequent mechanism of the targeted inhibition of MDSCs by the YPF formula.

With the deepening of MDSCs research, several signaling pathways were found involved in the regulation of MDSCs in recent years, such as the JAK/STAT3 pathway, NF-*κ*B pathway, PI3K/Akt pathway, MEK/ERK pathway, and so on. Among them, the activation of the JAK/STAT3 signaling pathway plays a crucial role in mediating tumor inflammatory response and promoting MDSCs proliferation [34, 35]. Sustained high expression of STAT3 could promote the proliferation of MDSCs and upregulate the expression of VEGF, thus promoting tumor development and angiogenesis [36]. These processes could eventually lead to continuous suppression of antitumor immunity and the adverse consequences of distant metastasis. The activation of the MEK/ERK signaling pathway could promote the recruitment of MDSCs into the tumor microenvironment, weaken the antitumor immune response, and allow tumor cells to evade immune surveillance and eradication [37, 38]. Furthermore, the immunosuppressive function of MDSCs is also associated with the activation of the PI3K/Akt signaling pathway [39]. In the present study, we detected the expression levels of key proteins involved in these signaling pathways. In vivo and in vitro experimental results suggested that the YPF formula could downregulate the expression levels of MDSCs proliferation-related proteins including phospho-STAT3, phospho-Akt, phospho-MEK, and phospho-ERK, thus leading to a marked decrease of MDSCs in the tumor microenvironment.

Based on the unique efficacy of the YPF formula in antilung cancer and limited drug resources, how to extract the maximum active ingredients and improve the utilization rate of the drug in the preparation of the YPF formula is also an urgent problem to be solved. It has been reported that some pretreatment methods, such as pre-soaking, liquid ammonia pretreatment, and hydrogen peroxide pre-soaking, have positive significance in stabilizing the chemical composition of drugs and maximizing the yield of drugs, which contributed to ensuring the clinical efficacy [40]. Water extraction and alcohol precipitation method, as a traditional process to achieve solid-liquid separation based on different solubility of drugs in water and ethanol, has been used in the preparation of the YPF formula for decades. A previous study indicated that the material-liquid ratio of 1 : 12, pre-soaking at 90°C for 1.5 h, 90% ethanol precipitation was the optimal process condition [41]. Besides, researchers have also explored the optimal preparation condition for ultrasonic extraction of the YPF formula. Results showed that the optimal condition was the material-liquid ratio of 1 : 20, the ultrasonic temperature of 55°C, and the ultrasonic time of 20 minutes. Compared with traditional extraction methods, ultrasound extraction has the characteristics of time-saving, energy-saving, and high efficacy, which provides the basis for large-scale production [42]. However, the final drug yields of both methods are still unsatisfactory, and better extraction methods need to be further explored [43].

To sum up, our study demonstrated that remodeling of the tumor microenvironment was one of the important mechanisms of the YPF formula's antilung cancer effect. The effect was manifested specifically in upregulating the positive immune cells and downregulating the negative immune cells. Among them, MDSCs played a critical role in the regulation process. By inhibiting proliferation and promoting apoptosis of MDSCs, the YPF formula reduced the levels of MDSCs in the tumor microenvironment. As a result, the oxidative stress was reduced and the negative regulatory effect on the proliferation of positive immune cells was removed, thus contributing to remodeling the tumor microenvironment.

## 5. Conclusions

The YPF formula can suppress subcutaneous xenograft growth of Lewis lung carcinoma tumor-bearing mice and prolong survival. Besides, the negative immune cells (MDSCs and its subsets, Treg) decrease, and the positive immune cells (CD_4_^+^*T* and CD_8_^+^ T lymphocytes) increase in vivo and in vitro. Among them, MDSCs play a crucial role in the immune regulation of the YPF formula. By promoting apoptosis and inhibiting the proliferation of MDSCs, the YPF formula achieves the effect of regulating the proportion of T-cell subsets and remodeling the tumor microenvironment.

## Figures and Tables

**Table 1:** The composition of the YPF formula.

Herb	Latin scientific name	Officinal part	Dosage (g)
Astragali radix (*Huang-Qi*)	*Astragalus membranaceus* (Fisch.) bunge	Root	18
Atractylodis Macrocephalae Rhizoma (*Bai-Zhu*)	*Atractylis macrocephala* (Koidz.) hand.-Mazz.	Rhizome	18
Saposhnikoviae Radix (*Fang-Feng*)	*Saposhnikovia divaricata* (Turcz.) Schischk.	Root	9

## Data Availability

The data used to support the findings of this study are included within the supplementary materials.

## Abbreviations

YPF: Yu-Ping-Feng
TCM: Traditional Chinese medicine
MDSCs: Myeloid-derived suppressor cells
TME: Tumor microenvironment
Treg: Regulatory T
AML: Acute myeloid leukemia
TAMs: Tumor-associated macrophages
HPLC: High performance liquid chromatography
NK: Natural killer
LLC: Lewis lung cancer
PI: Propidium iodide
PVDF: Polyvinylidene fluoride
SDS-PAG: Sodium dodecyl sulfate-polyacrylamide gel.

## Supplementary Materials

Supplementary Materials The granules of the YPF formula analyzed by HPLC, the purity of induced MDSCs cell populations determined by flow cytometry, the primary antibodies, dilutions, and source for Western blot are shown in the supplementary materials.
