# Supplementary material for: Yu-Ping-Feng Formula Exerts Antilung Cancer Effects by Remodeling the Tumor Microenvironment through Regulating Myeloid-Derived Suppressor Cells
Source: Evid Based Complement Alternat Med. 2021 Apr 20;2021:6624461. doi: 10.1155/2021/6624461 (PMC8079197; doi:10.1155/2021/6624461)
Supplement: Supplementary Materials — The granules of the YPF formula analyzed by HPLC, the purity of induced MDSCs cell populations determined by flow cytometry, the primary antibodies, dilutions, and source for Western blot are shown in the supplementary materials. [file 6624461.f1.docx]

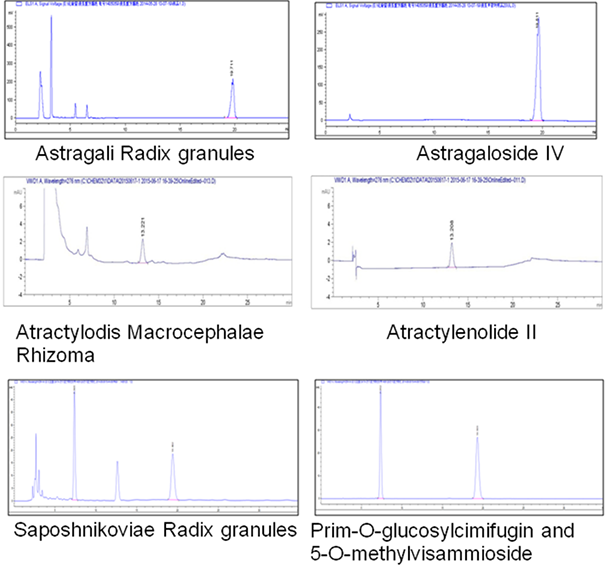


**Supplemental Figure 1:** The granules of the YPF formula were analyzed by HPLC. Among them, Astragaloside IV was used as the standard control of Astragali Radix granules (***Huang-Qi***). Atractylenolide II was used as the standard control of Atractylodis Macrocephalae Rhizoma (***Bai-Zhu***). Prim-O-glucosylcimifugin and 5-O-methylvisammioside were used as standard controls of Saposhnikoviae Radix granules (***Fang-Feng***).

**
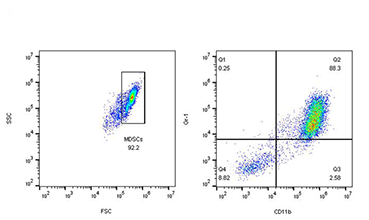
**

**Supplemental Figure2:** The purity of induced MDSCs cell populations determined by flow cytometry.

**Supplemental Table1** List of primary antibodies used in Western blot

| Antibody | host | Dilution | Source |
| --- | --- | --- | --- |
| STAT3 | Rabbit | 1:1000 | Cell Signaling Technology 12640 |
| p-STAT3 | Rabbit | 1:2000 | Cell Signaling Technology 9145 |
| AKT | Rabbit | 1:1000 | Cell Signaling Technology 4691 |
| p-AKT | Rabbit | 1:2000 | Cell Signaling Technology 4060 |
| MEK | Rabbit | 1:1000 | Cell Signaling Technology 8727 |
| p-MEK | Rabbit | 1:1000 | Cell Signaling Technology 9154 |
| ERK | Rabbit | 1:1000 | Cell Signaling Technology 4695 |
| p-ERK | Rabbit | 1:2000 | Cell Signaling Technology 4370 |
| β-actin | Rabbit | 1:1000 | Cell Signaling Technology 4970 |
